# Successful eculizumab treatment as an adjunctive therapy to desensitization in ABO-incompatible living donor kidney transplantation and its molecular phenotypes

**DOI:** 10.3389/fimmu.2024.1465851

**Published:** 2024-10-28

**Authors:** Ga Young Heo, Minsun Jung, Honglin Piao, Hyun Jeong Kim, Hyung Woo Kim, Juhan Lee, Kyu Ha Huh, Beom Seok Kim, Jaeseok Yang

**Affiliations:** ^1^ Department of Internal Medicine, College of Medicine, Yonsei University, Seoul, Republic of Korea; ^2^ Department of Pathology, College of Medicine, Yonsei University, Seoul, Republic of Korea; ^3^ Department of Surgery, College of Medicine, Yonsei University, Seoul, Republic of Korea

**Keywords:** ABO-incompatible kidney transplantation, antibody-mediated rejection, complement, desensitization, eculizumab

## Abstract

**Introduction:**

ABO-incompatible (ABOi) kidney transplantation (KT) has become an important option to overcome organ shortage. Plasmapheresis/rituximab-based desensitization therapy has successfully reduced anti-ABO antibody levels and suppressed antibody-mediated rejection (AMR) in ABOi KT. However, high titers of anti-ABO antibodies in some patients are refractory to standard desensitization, leading to loss of KT opportunities or AMR.

**Methods:**

Eculizumab treatment was used an adjunctive therapy to rescue high-titer ABOi KT patients refractory to plasmapheresis/rituximab-based desensitization. Molecular phenotypes of allograft biopsies and cellular phenotypes of peripheral blood mononuclear cells of eculizumab group were compared with those of control groups using the Banff Human Organ Transplant gene panel and flow-cytometric analysis, respectively.

**Results:**

The initial titers of anti-ABO antibodies in the two patients were 1:512 and >1:1024; the final pre-transplant titers after desensitization were 1:128 and 1:64. Both patients received eculizumab from KT day to two or four weeks post-KT and maintained stable renal function up to one-year post-transplantation without overt infection, despite early episodes of probable AMR or borderline T cell-mediated rejection. Molecular phenotype analysis revealed that gene expression patterns in the ABOi KT with eculizumab group overlapped with those in the ABOi KT with AMR group more than in the ABOi KT without AMR group, except for complement pathway-related gene expression. Anti-ABO antibody titers decreased to low levels 1–3 months post-transplant in the eculizumab group in parallel with decreasing anti-B-specific B cells.

**Conclusions:**

Short-term eculizumab therapy is promising for rescuing ABOi KT recipients with high anti-ABO antibody titers refractory to plasmapheresis-based desensitization therapy.

## 1 Introduction

ABO-incompatible (ABOi) living-donor kidney transplantation (KT) has increased rapidly with advancements in desensitization therapy (1). Recent desensitization treatment in ABOi KT aimed to eliminate existing antibodies and prevent anti-ABO antibody rebound produced by B cells (2). If the ABO antibody titers are effectively suppressed for the first 3-4 weeks after KT, a phenomenon called “accommodation” occurs, wherein rejection does not occur even if the anti-ABO antibody rebounds (3). On the other hand, high titers of anti-ABO antibodies at or after KT are associated with antibody-mediated rejection (AMR) (4, 5). The combination of plasmapheresis (PP) and anti-CD20 monoclonal antibody (rituximab), along with potent maintenance immunosuppressants such as tacrolimus and mycophenolate mofetil, has successfully reduced anti-ABO antibody titers and substantially improved the outcomes of ABOi KT (1, 2, 6). However, despite these improvements, desensitization fails to sufficiently decrease the titers of anti-ABO antibodies at the time of KT and cannot proceed to KT in some cases (3, 7). Although the percentages of patients who do not respond to traditional desensitization therapy are variable according to the target titer, previous studies have reported failure rates as high as 14-21% (1, 8, 9). In other cases, PP with intravenous immunoglobulin (IVIG) therapy cannot suppress the post-transplantation rebound of anti-ABO antibodies and subsequent AMR (3, 5).

Eculizumab (Soliris, Alexion Pharmaceuticals, New Haven, CT, USA), an anti-complement component 5 (C5) monoclonal antibody, inhibits C5 cleavage into C5a and C5b and the formation of the membrane attack complex C5b-9 (10). A previous randomized trial showed that eculizumab was a safe and effective option for preventing AMR in sensitized patients with anti-human leukocyte antigen (HLA) donor-specific antibodies (DSA) (11, 12). Moreover, our group demonstrated that C5 inhibitor-based immunosuppression induces accommodation in ABOi mouse heart transplantation (13). In parallel, eculizumab has been suggested as another desensitization option to prevent AMR in human ABOi KT (14).

Based on this background, we applied the eculizumab treatment as an adjunctive therapy to desensitization protocol to two cases of ABOi KT that maintained unacceptably high titers that were refractory to conventional PP-based desensitization at the time of transplantation.

## 2 Methods

### 2.1 Study population

This study enrolled two patients who underwent eculizumab treatment as an adjunctive therapy to desensitization for high-titer anti-ABO antibodies. To compare the molecular phenotypes of allograft biopsies, we included nine patients as controls: three patients with AMR in ABOi KT (AMR1 group), three patients without AMR in ABOi KT (no rejection [NR] group), and three patients with AMR in ABOi/HLA-incompatible (HLAi) KT (AMR2 group). The pathological findings were reviewed according to the 2022 Banff report (15). For the immunocellular phenotype analysis, we included three volunteers as healthy controls. This study was performed in accordance with the Declaration of Helsinki and the Declaration of Istanbul. This study was approved by the Institutional Review Board of Severance Hospital (4-2022-1265). Written informed consent was obtained from all the participants for study participation and publication of the details of their medical cases.

### 2.2 Treatment regimens

The two patients in the eculizumab group received a conjugated quadrivalent meningococcal vaccine (Menveo, Novartis Vaccines and Diagnostics, Cambridge, MA, USA) twice at two-month intervals, with the last dose administered two weeks before eculizumab administration. The patients also received chemoprophylaxis treatment with ciprofloxacin for a month during eculizumab administration. The standard desensitization protocol for ABOi KT consisted of rituximab (Mabthera, Roche Holding, Basel, Switzerland), PP, and low-dose (100 mg/kg) IVIG (IV-Globulin SN, Green Cross Corporation, Yongin, Korea). Induction therapy included an anti-interleukin-2 receptor monoclonal antibody (Basiliximab; Novartis Pharma AG, Basel, Switzerland) for ABOi KT patients and thymoglobulin for ABOi/HLAi KT recipients. Maintenance immunosuppressants consisted of a combination of corticosteroids, tacrolimus, and mycophenolate mofetil, initiated one week before KT. The eculizumab treatment as an adjunctive therapy to desensitization protocol for ABOi KT included eculizumab administration at one dose (1200 mg) before reperfusion, another dose (900 mg) on day 1, and a weekly dose (900 mg) for two or four weeks (**Supplementary Figure S1**). When PP was performed after eculizumab administration, 600 mg of eculizumab was additionally administered to replace the PP-related losses, according to a previous study (11). As previously reported, anti-ABO titers were determined by the tube test with serially diluting the patient’s serum using commercial A/B indicator red cells with 3.0% Affirmagen (Ortho Clinical Diagnostics, Raritan, NJ, USA). The ABO antibody titers were identified by determining the highest serum dilution ratio that showed a 1+ reactivity. Anti-ABO IgG titers were measured after incubation at 37°C, washing, and the addition of antihuman globulin for agglutination, while anti-ABO IgM titers were measured from untreated samples (16).

### 2.3 Banff human organ transplant gene panel

We analyzed immunological gene expression in kidney allograft biopsies using the B-HOT panel (NanoString, Seattle, WA, USA) (17). This panel was rigorously validated using formalin-fixed paraffin-embedded samples (18–20). RNA was extracted from formalin-fixed, paraffin-embedded tissues. One hundred nanograms of RNA from each biopsy was added to the NanoString codeset in a hybridization buffer and incubated at 65°C for 16 h. Gene expression analysis was performed according to the standard protocols recommended by the manufacturer (NanoString Technologies, Seattle, WA, USA). Expression of isolated transcripts was assayed using the nCounter SPRINT system (NanoString Technologies, Seattle, WA, USA). Data preprocessing and quality control were performed according to the standard protocols provided by the manufacturer using nSolver software version 4.0, NanoStringQCPro version 1.14.0, and NanoStringNorm version 1.2.1 (NanoString Technologies). Gene counts were normalized using the geometric mean of the internal reference genes, and gene expression was log-2 transformed.

### 2.4 Gene sets and gene expression analysis

Cell-type profiling and microarray-based pathway scoring were conducted using nCounter Advanced Analysis ver. 2.0 (NanoString). The first principal component (PC) of the gene sets corresponds to the microarray-based pathway scores (18, 19). Gene set annotations were retrieved from published studies (17, 19, 21, 22). We also calculated the gene set variation analysis (GSVA) enrichment scores of individual samples for the msigdb hallmark gene sets using a GSVA package, as previously applied to the B-HOT assay (20), after curation of the gene symbols (**Supplementary Tables S1**, **S2**). Differential gene set enrichment was identified with a two-sided t-test P-value <0.05. Differentially expressed genes (DEGs) were determined using a two-sided t-test at a threshold of |fold-change| ≥ 1.5 and a false discovery rate (FDR) < 0.05. Functional enrichment of differentially expressed genes (DEGs) was annotated using Gene Ontology (GO), Reactome, and WikiPathway databases (23–26). Visualization of the allograft rejection pathway in WikiPathway was conducted using Cytoscape ver. 3.8.2 (26–28).

### 2.5 Cellular immunophenotypic analysis

Peripheral blood mononuclear cells were prepared by density gradient separation using Ficoll (GE Healthcare, Cleveland, OH, USA) and stained for CD4, CD19, CD27, CD43, CXCR5, PD-1, IFN-γ, IL-4, IL-17, IL-10, and Foxp3 using fluorochrome-labeled antibodies (**Supplementary Table S3**). The frequency of blood group B-specific B cells was measured using a blood group B antigen-polyacrylamide-fluorescein isothiocyanate complex (GlycoNZ, New Zealand). For intracellular cytokine staining, isolated cells were incubated with 500 ng/mL of ionomycin (Merk, Pahway, NJ, USA) and 50 ng/mL of phorbol myristate acetate and GolgiStop (BD Biosciences, CA, USA) at 37°C for 5 hours. For intracellular Foxp3 staining, the cells were fixed and permeabilized using Foxp3/Transcription Factor Staining Buffer Set (Thermo Fisher Scientific, Waltham, MA, USA). Flow cytometric analysis was performed using an Attune NxT flow cytometer (Thermo Fisher Scientific) and FlowJo software (Tree Star, Ashland, OR, USA).

## 3 Results

### 3.1 Clinical course of case 1 in the eculizumab group

**Figure 1:**
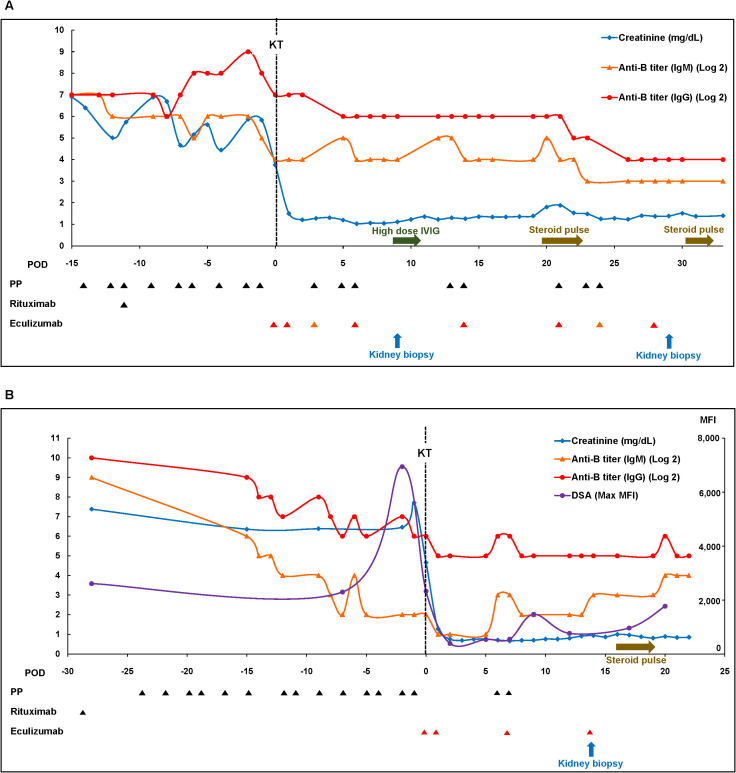
Clinical courses of patients in the eculizumab group. **(A)** Case 1 and **(B)** Case 2. Max, maximal; MFI, mean fluorescence intensity; PP, plasmapheresis.

A 67-year-old man with blood type O underwent KT for diabetic end stage kidney disease (ESKD) from his wife with blood type B. His baseline clinical characteristics are presented in **Supplementary Table S4**. Eight months prior, the patient had undergone desensitization therapy involving rituximab administration and PP; however, KT was canceled, because anti-B IgG titer failed to decrease to <1:128. Therefore, we decided to receive eculizumab treatment as an adjunctive therapy to desensitization. Baseline IgM and IgG anti-B antibody titers were 1:512 and 1:512, respectively. The lymphocyte crossmatch test results were negative, with no DSA. The patient received rituximab and underwent PP with the replacement of low-dose IVIG. After six sessions of PP with IVIG, we observed a rebound in the anti-B IgG titer, and the final preoperative anti-B titer was 1:128 on day 1 (**Table 1**, **Figure 1A**). Eculizumab was administered two hours before KT, on postoperative day 1, and weekly for four weeks (days 6, 14, 21, and 28). There was no evidence of hyperacute rejection. Because he showed slightly reduced urine output, anemia, and thrombocytopenia with an IgG anti-B of 1:128 on day 3, we performed three sessions of PP. Additional doses of eculizumab were administered to replace the PP-related loss on days 3 and 24. A kidney biopsy on day 9 revealed mild glomerulitis, suggesting the possibility of active AMR. High-dose IVIG treatment and two sessions of PP were performed for increasing serum creatinine levels with elevated anti-B titers (1:64). However, the serum creatinine level increased to 1.80 mg/dL on day 20, and we administered 3 days of empirical steroid pulse therapy and three sessions of PP. A follow-up kidney biopsy on day 29 showed borderline T cell-mediated rejection (TCMR) along with peritubular capillaritis, which was treated by 2 days of steroid pulse therapy. The patient’s renal function stabilized without further treatment on day 24 (**Figure 1A**). Serum levels of C3 and C4 were maintained within normal ranges, and the CH50 (50% hemolytic complement), which was <10% during eculizumab therapy, had returned to the normal range three months after KT (**Table 1**). No infection-related complications occurred up to one year after KT. Serum creatinine levels were stable (1.33 mg/dL) and anti-B titer levels were low (1:16) one-year post-KT (**Table 1**).

**Table 1:** Changes of anti-ABO antibody titers and laboratory findings of patients in the eculizumab group.

Case	Variable	Pre-transplant period		Post-transplant period						
		Pre-PP	Post-PP^a^	1 day	7 day	1M	2M	3M	6M	12M
Case 1	Serum creatinine (mg/dL)	6.91	2.36	1.65	1.03	1.37	1.33	1.34	1.21	1.33
	Anti-ABO Titers, (IgM/IgG)	1:512/1:512	1:16/1:128	1:16/1:128	1:64/1:64	1:8/1:16		1:8/1:8		1:16/1:16
	C3/C4 (mg/dL)				70.3/ 13.34	83.6/ 26.35	89.6/ 26.18	95.5/ 24.77		94.7/ 23.06
	CH50 (U/mL)				<10	<10	57.5	68.4		29.1
	DSA (MFI)	None	None					None		None
	UPCR (g/gCr)	2.76	2.03	1.85	4.27	0.81	0.24	0.33	0.17	0.19
Case 2	Serum creatinine (mg/dL)	7.24	7.71	1.28	0.69	0.91	0.99	0.79	0.90	0.80
	Anti-ABO Titers, (IgM/IgG)	1:256/>1:1024	1:4/1:64	1:2/1:32	1:8/1:64	1:16/1:32	1:32/1:64	1:16/1:32	1:16/1:32	1:16/1:64
	C3/C4 (mg/dL)			64.8/10.22	76.7/ 12.07	81.6/ 23.24	101.6/27.10	94.1/ 23.64	98.4/ 24.69	100.8/ 25.93
	CH50 (U/mL)			1.6	1.0	3.4	60.4	54.8	57.9	58.7
	DSA, MFI	B54 (2607) A33 (1474)	B54 (2327)	B54 (391)	B54 (548)	B54 (4589) A33(606) B46 (237)	B54 (1630)	B54 (628)	B54 (404)	B54(346)
	UPCR (g/gCr)	5.71	2.61	1.22	0.45	0.26	0.20	0.10	0.08	0.11

PP, plasmaphresis; M, month; IgM, Immunoglobulin M; IgG, Immunoglobulin G; C3, complement component 3; C4, complement component 4; CH50, 50% hemolytic complement; DSA, donor-specific antibodies; MFI, mean fluorescence intensity; UPCR, urine protein to creatinine ratio.

a Post-PP time refers to the one day before transplantation.

### 3.2 Clinical course of case 2 in the eculizumab group

A 48-year-old woman with blood type O underwent KT for diabetic ESKD from a nephew with blood type B. Her baseline clinical characteristics are presented in **Supplementary Table S4**. The pretreatment IgM and IgG anti-B Ab titers were 1:256 and >1:1024, respectively. Although the results of complement-dependent cytotoxicity crossmatch were negative, flow-cytometric crossmatch (FCX) results were positive; the median channel shift of T cell FCX was 227, while that of B cell FCX was 267. DSA was positive for B54 (mean fluorescence intensity [MFI]: 2,607) and A33 (MFI: 1,474). After rituximab and 14 sessions of PP, T-cell FCX results were negative, with a low titer of anti-B54 DSA (MFI: 2,327). The IgM anti-B titer successfully decreased to 1:4; however, the IgG anti-B titer remained high (1:64) (**Table 1**, **Figure 1B**). To proceed with KT, we administered eculizumab before KT and on days 1, 7, and 14. The patient had decreased serum creatinine levels without any evidence of hyperacute rejection. Because the post-KT anti-B titer increased to 1:64 on day 5, two sessions of post-KT PP were performed to prevent AMR. A biopsy on day 14 revealed peritubular capillaritis, which raised the possibility of active AMR and led to steroid pulse therapy (methylprednisolone 250 mg). The patient was discharged after confirming that the serum creatinine level had improved and that the anti-B titer was maintained at 1:32 (**Figure 1B**). Although she did not experience major infectious complications, the whole-blood PCR titer of the BK increased to 277,000 copies/mL three months post-KT. The tacrolimus dose was reduced from 4 mg to 2.5 mg, and mycophenolate mofetil was replaced with sirolimus. The CH50 was <10% during eculizumab therapy and returned to the normal range two months after KT. Serum creatinine levels remained stable (0.80 mg/dL) one-year post-KT. The MFI of the anti-B54 DSA titer and the anti-B titer was 346 and 1:64, respectively, one-year post-KT (**Table 1**).

### 3.3 Transcriptomic analysis using the B-HOT gene panel

We compared the transcriptomic profiles of 12 biopsies obtained from case 1 (two biopsies) and 2 (one biopsy) in the eculizumab group (n=3) and control groups, including AMR1 (n=3), NR (n=3), and AMR2 (n=3). The baseline characteristics of nine patients in control groups are presented in **Supplementary Table S5**. Pathological details are shown in **Supplementary Table S6**. A total of 758 endogenous genes were quantified (**Supplementary Table S7**). The samples showed an intercorrelation (r = 0.8), whereas the first (biopsy 1-1, day 9) and second (biopsy 1-2, day 29) biopsies from case 1 did not exhibit a notably stronger association (**Supplementary Figure S2**). However, these two biopsies showed differing transcriptomic profiles, with immune-related genes upregulated in biopsy 1-2 (**Supplementary Figure S3**), concurring with greater histological inflammation (**Supplementary Table S6**).

#### 3.3.1 Comparison between the eculizumab group and the AMR1 group

**Figure 2:**
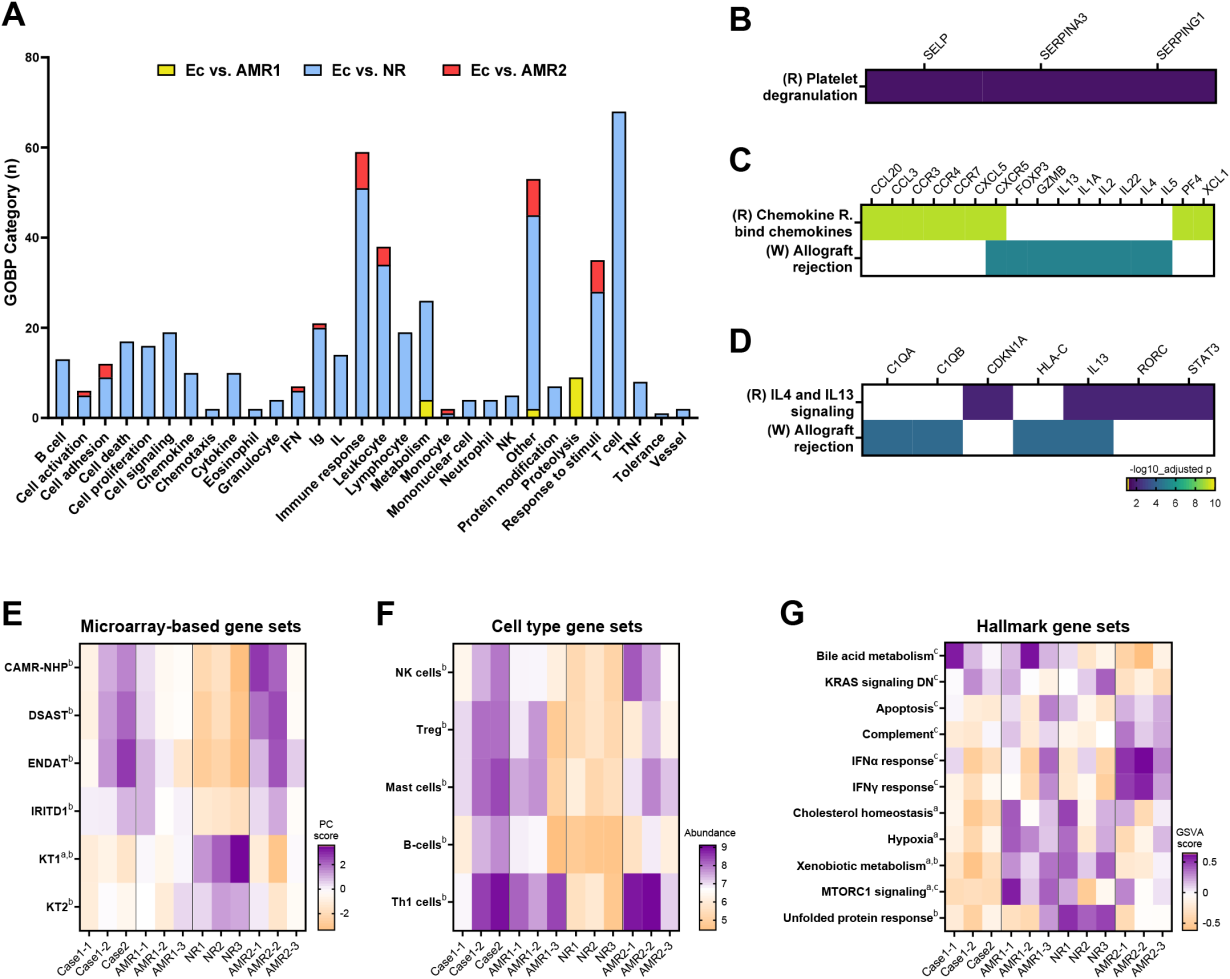
Transcriptomic characteristics of the eculizumab group. **(A)** Gene ontology-biologic process analysis of differentially expressed genes of the Ec group compared to the AMR1, NR, and AMR2 groups. **(B)** Platelet degranulation pathway enriched between Ec and AMR1 groups. **(C)** Chemokine and allograft rejection pathways enriched between Ec and NR groups. **(D)** IL-4/-13 signaling and allograft rejection pathways enriched between Ec and AMR2 groups. **(E)** Diagnostic gene sets reported from microarray-based studies enriched in the Ec group compared to the other groups. **(F)** Cell-type abundance differs between the Ec group and the other groups. **(G)** GSVA scores differ between the Ec group and the other groups. Superscripts denote P-values <0.05 in the following comparison: **(A)**, Ec vs. AMR1; **(B)**, Ec vs. NR; **(C)**, Ec vs. AMR2. Heapmaps are row-clustered. AMR1, ABO-incompatible kidney transplantation group with antibody-mediated rejection; AMR2 ABO-incompatible and HLA-incompatible kidney transplantation group with antibody-mediated rejection; Ec, eculizumab group; NR, ABO-incompatible kidney transplantation group without antibody-mediated rejection.

In comparing the eculizumab group with the AMR1 group, 11 DEGs were identified (**Figure S4**). Among these, SERPINA3 was downregulated, whereas *IL27* and *IL17F* were upregulated in the eculizumab group. Functional enrichment analysis revealed enrichment in proteolysis and metabolism in the eculizumab group compared to the AMR1 group, suggesting altered metabolic activity (**Figure 2A**; **Supplementary Table S8**). Additionally, pathway analysis showed differential enrichment in platelet degranulation in the eculizumab group compared to the AMR1 group (**Figure 2B**). This indicates a possible involvement of altered platelet activity in the eculizumab-treated patients.

#### 3.3.2 Comparison between the eculizumab group and the NR group

In comparing the eculizumab group with the NR group, 114 DEGs were identified (**Supplementary Figure S4**). Among these, IL27 and IL17F were significantly upregulated in the eculizumab group, highlighting a distinct immune response. Functional enrichment analysis revealed that the eculizumab group exhibited enrichment in diverse immunological pathways compared to the NR group (**Figure 2A**, **Supplementary Table S8**). Pathway analysis showed differential enrichment in chemokine signaling and allograft rejection pathways in the eculizumab group compared to the NR group (**Figure 2C**). Allograft rejection, which was enriched in the eculizumab group compared to the NR groups, was differentially affected at the gene level. Additionally, T cell-related genes were upregulated in the eculizumab group, while no alterations in MHC or complement genes were observed, indicating a specific impact on T cell activity without affecting MHC or complement pathways. In addition, microarray-based gene sets related to chronic antibody-mediated rejection (CAMR), donor-specific antibody (DSAST), endothelium (ENDAT), and injury/repair (IRITD1) were upregulated, whereas those related to renal tubulointerstitium (KT) were downregulated in the eculizumab group compared to those in the NR group (**Figure 2E**). Cell-type profiling indicated an increased abundance of NK cells, regulatory T cells, mast cells, B cells, and Th1-cells in the eculizumab group compared to the NR group (**Figure 2F**).

#### 3.3.3 Comparison between the eculizumab group and the AMR2 group

**Figure 3:**
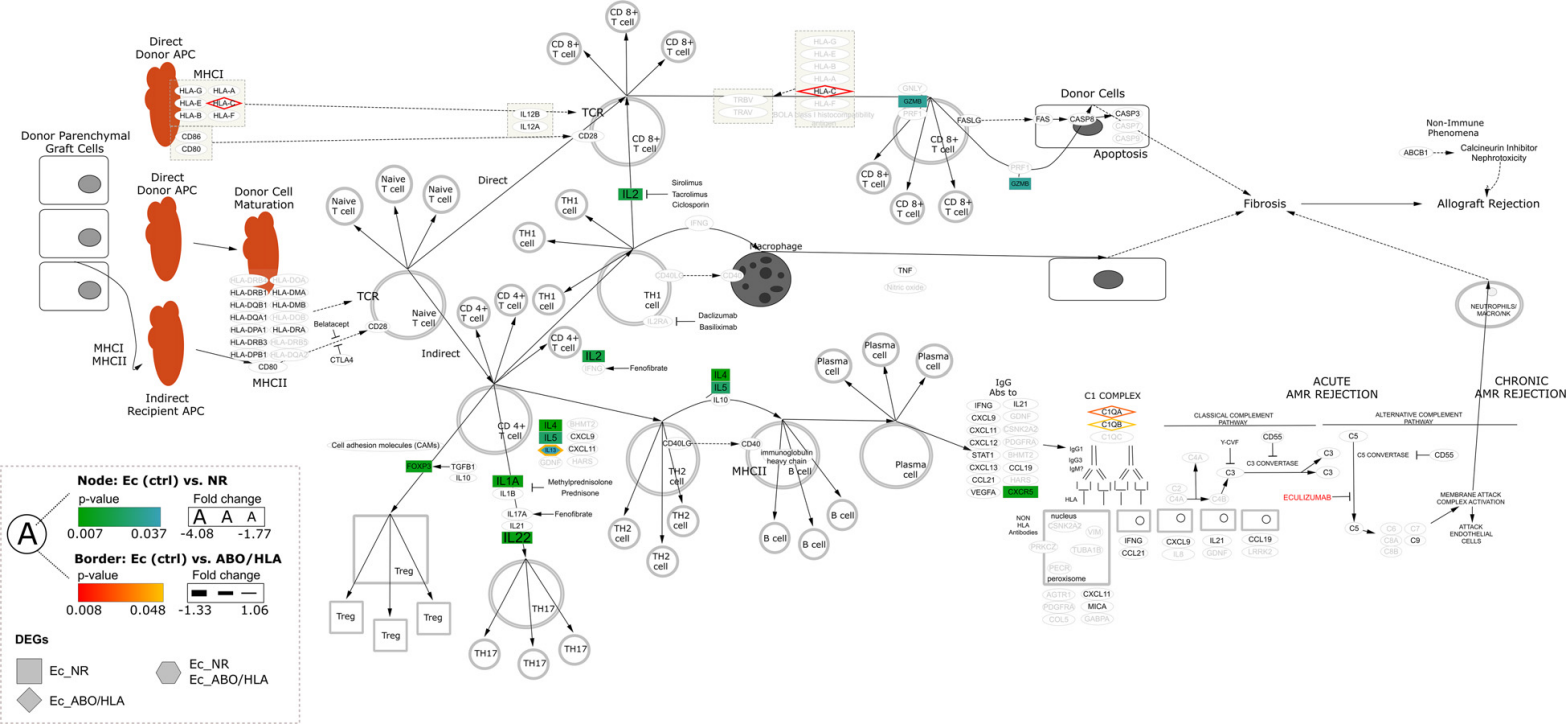
Differentially expressed genes of the Ec group compared to the NR and AMR2 group that were enriched for the allograf rejection pathway (WikiPathway: WP2328). DEG groups, with significance levels and fold change, are presented in the legends (left lower corner). B-HOT genes not included in DEGs are written in black with gray. AMR1, ABO-incompatible kidney transplantation group with antibody-mediated rejection; AMR2 ABO-incompatible and HLA-incompatible kidney transplantation group with antibody-mediated rejection; APC, antigen-presenting cell; B-HOT, Banff Human Organ Transplant; DEG, differentially expressed genes; Ec, eculizumab group; HLA, human leukocyte antigen; IL, interleukin; MHC, major histocompatibility complex; NR, ABO-incompatible kidney transplantation group without antibody-mediated rejection; PCA, principal component analysis.

When comparing the eculizumab group with the AMR2 group, 21 DESs were detected (**Supplementary Figure S4**). Notably, HLA-C was significantly downregulated in the eculizumab group compared to the AMR2 group. Functional enrichment analysis indicated that immune response and leukocyte-related functions were more prominent in the eculizumab group, pointing to heightened immune activity in comparison to the AMR2 group (**Figure 2A**; **Supplementary Table S8**). Pathway analysis identified distinct enrichment in interleukin-4/13 signaling pathways and allograft rejection processes in the eculizumab group relative to the AMR2 group (**Figure 2D**). Furthermore, allograft rejection, which was more pronounced in the eculizumab group, showed gene-level differences, with HLA-C, C1QA, and C1QB downregulated compared to the AMR2 group (**Figure 3**). In addition, hallmark gene set analysis highlighted a significant downregulation of the complement pathway in the eculizumab group compared to the AMR2 group (**Figure 2G**).

### 3.4 Cellular immunophenotypic analysis

The health control groups have a mean age of 30 years, consisting of two donors with blood type O and one with blood type A, all without any comorbidities. Proportions of IFN-γ^+^, IL-4^+^, and IL-17^+^ CD4^+^ T cells, and CXCR5^+^PD-1^+^CD4^+^ follicular helper T cells in peripheral blood seemed to increase after post-transplant two weeks to three months in case 2 compared to case 1 and the healthy controls, whereas those of Foxp3^+^CD4^+^ regulatory T cells and IL-10^+^CD4^+^ T cells showed transient increase at two weeks in case 2 (**Supplementary Figures S5A–F**). The proportion of anti-B-specific B cells was elevated for 1–3 months after KT and then decreased to basal levels in the eculizumab group (**Supplementary Figure S5G**). The proportion of IL-10^+^ B cells was transiently elevated in the eculizumab group compared to that in the healthy controls (**Supplementary Figure S5H**).

## 4 Discussion

This study showed that short-term eculizumab therapy over 2–4 weeks led to successful ABOi KT with unacceptably high anti-ABO antibody titers refractory to PP-based standard desensitization and stable allograft functions without significant complications up to one-year post-transplant. The eculizumab group showed immunologically activated molecular phenotypes compared to the ABOi group without AMR and shared many features of the molecular phenotypes of the ABOi and ABOi/HLAi groups with AMR, except for complement activation pathway-related genes.

A single-center study reported that eculizumab reduced the incidence of early AMR in crossmatch-positive HLAi KT recipients (29). Moreover, in a phase 2 randomized controlled trial, an eculizumab protocol showed a lower incidence of AMR in one-year protocol biopsies than the standard PP-based desensitization protocol in HLAi KT (11). Anti-ABO antibodies also activate the complement system, and the deposition of C4d in peritubular capillaries is common in ABOi KT (30). Considering the important role of complement activation in AMR in ABOi KT, a nine-week course of eculizumab therapy was attempted in addition to PP-based desensitization in four cases of ABOi KT (14). However, the initial (1:64, 1:64, 1:16) and final (1:32, 1:32, 1:4) anti-ABO titers before KT were not high in three patients, and one patient with a high initial pre-transplant titer (1:2,048) had a low final pre-transplant anti-A titer (1:2) and a blood group A2 mismatch with the donor, with a much lower risk of AMR than an A1 mismatch (31). In contrast to the low-risk profiles in the previous study, patients in the present study had high initial (1:256, 1:1,024) and final (1:128, 1:64) anti-B titers before KT, which are refractory to PP-based desensitization. Therefore, this study clearly showed the promising role of eculizumab treatment as an adjunctive therapy to desensitization in the very high-risk group with unacceptably high anti-ABO titers compared to standard desensitization.

In this study, we gave induction eculizumab dose on day 0, weekly maintenance doses, and additional replacement dose of eculizumab after plasmapheresis according to the previous protocol (11, 12). Regarding the duration of eculizumab therapy, we administered short courses of eculizumab for two or four weeks instead of nine weeks as in previous studies (11, 12, 14) because accommodation is usually established two to three weeks after ABOi KT, and the anti-ABO antibody does not increase the risk of chronic AMR in contrast to anti-HLA DSA (3, 32). The successful outcomes of short-course eculizumab therapy in this study support the relative benefits of short-term eculizumab therapy in ABOi KT over a nine-week course of eculizumab therapy in HLAi KT, considering the potential infectious complications and the high cost of prolonged eculizumab therapy (33).

Anti-B antibody titers decreased to low levels 1–3 months post-transplant in the eculizumab group in parallel with decreasing anti-B-specific B cells at this time point, suggesting that declining anti-donor ABO-specific B cells could contribute to the accommodation of anti-ABO antibody responses (34, 35). In the ABOi/HLAi case, anti-HLA B54 DSA levels were also maintained at low levels up to one-year post-transplant in parallel with low proportions of circulating follicular helper T cells at this time point (36).

Transcriptomic analysis demonstrated that the eculizumab group had more immunostimulatory phenotypes than the NR group, similar to the AMR groups. However, the eculizumab group showed only borderline histology of AMR and TCMR and had lower expression of complement pathway-related genes than the AMR groups, suggesting that terminal blockade of the complement pathway by eculizumab may protect this group from overt allograft rejection in contrast to the AMR groups.

This study had several limitations. First, we applied the new short-term eculizumab treatment as an adjunctive therapy to desensitization protocol to only two patients with a one-year follow-up. Based on this pilot study, further large-scale, prospective studies with longer follow-up periods are needed to confirm the effectiveness and safety of this regimen in patients undergoing ABOi KT. Second, adjunctive eculizumab treatment following desensitization did not completely prevent occurrence of antibody-mediated rejection. However, degree of antibody-mediated rejection was mild (‘probable antibody-mediated rejection’) despite high post-operative anti-ABO titer (1:64-1:128), and probable antibody-mediated rejections in both cases were successfully managed by eculizumab and other treatment (plasmapheresis, IVIG, or steroid), with good renal functions up to post-transplant 1 year. Furthermore, it is difficult to attribute the efficacy of eculizumab, given that we combined PP, IVIG, or steroid pulse therapy empirically based on the small changes in clinical features and laboratory findings, especially in case 1. However, molecular phenotypes of the eculizumab group showed lower expression of complement pathway-related genes and this distinct profile suggests that the blockade of the complement pathway by eculizumab may have contributed to the observed good outcomes independently of other treatments. Further prospective controlled studies are required to elucidate the necessity of post-transplant PP and independent roles of eculizumab in an adjunctive eculizumab treatment to desensitization protocol.

Nevertheless, to our knowledge, this study is the first to show that short-term eculizumab treatment as an adjuctive therapy to desensitization therapy can lead to successful ABOi KT with good renal allograft function and tolerable safety profiles during the first year in high-risk patients with unacceptably high anti-ABO antibody titers refractory to standard PP-based desensitization. Furthermore, cellular and molecular phenotypic analyses provided mechanistic insights into this regimen in ABOi KT recipients.

In conclusion, short-term eculizumab treatment as an adjunctive therapy to desensitization therapy is a promising strategy for ABOi KT recipients with high anti-ABO antibody titers.

## Data Availability

The datasets presented in this study can be found in online repositories. The names of the repository/repositories and accession number(s) can be found below: GSE274052 (GEO).
