# Supplementary material for: Successful eculizumab treatment as an adjunctive therapy to desensitization in ABO-incompatible living donor kidney transplantation and its molecular phenotypes
Source: Front Immunol. 2024 Oct 28;15:1465851. doi: 10.3389/fimmu.2024.1465851 (PMC11550984; doi:10.3389/fimmu.2024.1465851)
Supplement: Supplementary file 1 [file DataSheet1.docx]

**Supplementary Materials**

**List of Supplementary Materials**

**TABLE S1.** Gene conversion data

**TABLE S2.** Analyzed gene sets

**TABLE S3.** Information of antibodies used in flow-cytometric analysis

**TABLE S4.** Baseline clinical characteristics of patients in the eculizumab group

**TABLE S5.** Baseline clinical characteristics of patients in control groups

**TABLE S6.** Pathologic characteristics of renal allograft biopsies

**TABLE S7.** Normalized gene expression counts across the samples

**TABLE S8.** Functional enrichment analysis

**FIGURE S1.** Regimen of an adjunctive eculizumab therapy and desensitization

**FIGURE S2.** Correlation analysis and principal component analysis

**FIGURE S3.** The top 5 Gene Ontology terms enriched in differentially expressed genes upregulated in the (**A**) first (biopsy 1-1) and (**B**) second (biopsy 1-2) biopsies of case 1

**FIGURE S4.** Differentially expressed genes between the (**A**) Ec and AMR1 groups, (**B**) Ec and NR groups, and (**C**) Ec and AMR2 groups

**FIGURE S5.** Changes in immune cell subsets during the post-transplant first year

**TABLE S1.** Gene conversion data.

See a separate excel file.

**TABLE S2.** Analyzed gene sets.

See a separate excel file.

**TABLE S3.** Information of antibodies used in flow-cytometric analysis

| Antibody or antigen | Fluorochrome | Clone | Target cells | Manufacturer | Catalog number |
| --- | --- | --- | --- | --- | --- |
| CD4 | FITC | OKT4 | Subsets of T cell | Biolegend | 317408 |
| IFN-γ | FITC | 4S.B3 | Activated T cell | Biolegend | 502506 |
| IL-4 | PE-cy7 | MP4-25D2 | Activated T helper 2 cell | Biolegend | 500824 |
| IL-17A | APC-cy7 | BL168 | Activated T cell | Biolegend | 512320 |
| CXCR5 (CD185) | APC | J252D4 | Follicular helper T cell | Biolegend | 356908 |
| PD-1 (CD279) | FITC | NAT105 | Activated T cell | Biolegend | 367412 |
| Foxp3 | PE | PCH101 | Regulatory T cell | invitrogen | 12-4776-42 |
| IL-10 | APC | JES3-9D7 | Regulatory T cell, Regulatory B cell | BD Biosciences | 130-096-042 |
| CD19 | APC | HIB19 | B cells | Invitrogen | 17-0199-42 |
| CD20 | V450 | L27 | B cells | BD Biosciences | 561164 |
| B antigen | FITC | Btri-C3-PAA | Anti-B-specific B cell | GlycoNZ | 0086-FP |

APC, allophycocyanin; FITC, fluorescein; Foxp3, forkhead box P3; IFN, interferon; IL, interleukin; PD-1, programmed cell death protein-1; PE, phycoerythrin.

**TABLE S4.** Baseline clinical characteristics of patients in the eculizumab group

| Variables | Case 1 | Case 2 |
| --- | --- | --- |
| Age, years | 68 | 48 |
| Sex | M | F |
| Cause of ESKD | Diabetic nephropathy | Diabetic nephropathy |
| Dialysis modality | HD | HD |
| Duration of dialysis, months | 9 | 17 |
| Recipient blood type | O | O |
| Donor blood type | B | B |
| HLA mismatch number | 5 | 6 |
| A, B | 3 | 4 |
| DR | 2 | 2 |
| DQ | 2 | 2 |
| DSA, MFI | None | B54(2327) |
| Desensitization treatment |  |  |
| Preoperative plasmapheresis | Yes | Yes |
| Preoperative rituximab | Yes | Yes |
| Maintenance Immunosuppression |  |  |
| Calcineurin inhibitor | Tacrolimus | Tacrolimus |
| Antimetabolite | Mycophenolate mofetil | Mycophenolate mofetil |
| Tacrolimus trough level, ng/mL | 6.7 | 2.8 |
| Donor age, years | 69 | 28 |
| Donor sex | Female | Male |
| Donor relation | Wife | Nephew |

ESKD, end stage kidney disease; HD, hemodialysis; HLA, human Leukocyte Antigen; DSA, donor-specific antibodies; MFI, mean fluorescence intensity

**TABLE S5.** Baseline clinical characteristics of patients in control groups

| Variables | ABOi KT with AMR (AMR1) | | | ABOi KT without AMR (NR) | | | ABOi HLAi KT with AMR (AMR2) | | |
| --- | --- | --- | --- | --- | --- | --- | --- | --- | --- |
|  | AMR1-1 | AMR1-2 | AMR1-3 | NR1 | NR2 | NR3 | AMR2-1 | AMR2-2 | AMR2-3 |
| Age, years | 51 | 57 | 62 | 58 | 42 | 68 | 54 | 53 | 56 |
| Sex | Femal | Male | Female | Female | Male | Male | Female | Female | Female |
| Cause of ESRD | IgA nephropathy | Diabetic nephropathy | Hypertensive nephrosclerosis | IgA nephropathy | IgA nephropathy | Polycystic kidney | unknown | Hypertensive nephrosclerosis | Unknown |
| Dialysis modality | HD | HD | HD | Preemptive | Preemptive | Preemptive | PD | HD | Preemptive |
| Dialysis duration, mons | 10 | 4 | 10 | NA | NA | NA | 27 | 2 | NA |
| Recipient blood type | B | O | A | O | A | O | O | O | B |
| Donor blood type | A | B | AB | A | B | B | A | A | B |
| HLA mismatch number | 5 | 5 | 6 | 5 | 5 | 5 | 4 | 4 | 6 |
| Desensitization |  |  |  |  |  |  |  |  |  |
| Plasmapheresis | Yes | Yes | Yes | Yes | Yes | Yes | Yes | Yes | Yes |
| Rituximab | Yes | Yes | Yes | Yes | Yes | Yes | Yes | Yes | Yes |
| Maintenance Immunosuppression |  |  |  |  |  |  |  |  |  |
| Calcineurin inhibitor | Tacrolimus | Tacrolimus | Tacrolimus | Tacrolimus | Tacrolimus | Tacrolimus | Tacrolimus | Tacrolimus | Tacrolimus |
| Antimetabolite | Mycophenolate mofetil | Mycophenolate mofetil | - | Mycophenolate mofetil | Mycophenolate mofetil | Mycophenolate mofetil | Mycophenolate mofetil | Mycophenolate mofetil | - |
| Tacrolimus level, (ng/mL) | 6 | 3.4 | 8 | 4.4 | 9.3 | 6.1 | 8.8 | 3.5 | 2..3 |
| Donor age, years | 54 | 55 | 61 | 63 | 38 | 58 | 20 | 57 | 59 |
| Donor sex | Male | Female | Male | Male | Female | Female | Female | Male | Male |
| Donor relation | LURD | LURD | LURD | LURD | LURD | LURD | LRD | LURD | LURD |
| Biopsy date,  postoperative day | 9 | 19 | 12 | 40 | 43 | 31 | 42 | 21 | 30 |
| eGFR, mL/min/1.73 m^2^  on biopsy day | 32 | 31 | 20 | 50 | 49 | 54 | 67 | 51 | 15 |
| Anti-ABO titers (IgM/IG) |  |  |  |  |  |  |  |  |  |
| Pre-desensitization | 1:16/1:64 | 1:32/1:128 | 1:8/1:2 | 1:4/1:64 | 1:4/1:2 | 1:2/1:16 | 1:16/1:128 | 1:8/1:64 | 1:64/1:256 |
| On operation day | 1:4/1:16 | 1:4/1:16 | 1:1/1:2 | 1:1/1:8 | 1:4/1:2 | 1:1/1:4 | 1:2/1:16 | 1:2/1:8 | 1:2/1:4 |
| On biopsy day | 1:4/1:64 | 1:8/1:16 | 1:1/1:2 | 1:1/1:4 | 1/64/1:16 | 1:2/1:8 | 1:4/1:16 | 1:8/1:64 | 1:1/1:2 |
| Donor-specific antibody |  |  |  |  |  |  |  |  |  |
| On operation day | none | none | none | none | none | none | A24 (402) DR52 (1325) | DR4 (1511) | B44 (1947)  DR4 (1041) DQ4 (2656) |
| On biopsy day | none | none | none | none | none | none | A24 (328) DR52 (6090) | DR4 (1850) | B44 (7769)  DR4 (4495)  DQ4 (1330) |

ABOi, ABO-incompatible; AMR, antibody-mediated rejection; eGFR, estimated glomerular filtration rate; ESRD, end stage renal disease; HD, hemodialysis; HLA, human leukocyte antigen; HLAi, HLA-incompatible; mons, months; LURD, living-unrelated donor; NA, not applicable; NR, no rejection; PD, peritoneal dialysis.

**TABLE S6.** Pathologic findings of renal allograft biopsies

| Banff score | Eculizumab | | | ABOi KT with AMR (AMR1) | | | ABOi KT without AMR (NR) | | | ABOi HLAi KT with AMR (AMR2) | | |
| --- | --- | --- | --- | --- | --- | --- | --- | --- | --- | --- | --- | --- |
|  | Case1-1 | Case1-2 | Case2 | AMR1-1 | AMR1-2 | AMR1-3 | NR1 | NR2 | NR3 | AMR2-1 | AMR2-2 | AMR2-3 |
| Histological diagnosis | Probably active AMR | Borderline TCMR | Probably active AMR | Active AMR | Probably active AMR | Active AMR | No rejection | No rejection | No rejection | Active AMR, borderline TCMR | Active AMR | Active AMR |
| Number of glomeruli | 7 | 9 | 18 | 14 | 6 | 7 | 23 | 9 | 11 | 14 | 9 | 12 |
| g | 1 | 0 | 0 | 2 | 1 | 0 | 0 | 0 | 0 | 1 | 3 | 1 |
| ptc | 0 | 2 | 1 | 1 | 0 | 1 | 0 | 0 | 0 | 3 | 2 | 3 |
| v | 0 | 0 | 0 | 1 | 0 | 2 | 0 | 0 | 0 | 1 | 0 | 0 |
| C4d | 2 (IHC) | 2 (IHC) | 3 (IHC) | 3 (IF) | 3 (IHC) | 1 (IHC) | 1 (IF) | 1 (IHC) | 0 (IHC) | 2 (IHC) | 2 (IF) | 3 (IF) |
| cg | 0 | 0 | 0 | 0 | 0 | 0 | 0 | 0 | 0 | 0 | 0 | 1 |
| ct | 0 | 0 | 0 | 0 | 0 | 0 | 1 | 0 | 0 | 0 | 0 | 0 |
| ci | 0 | 0 | 0 | 0 | 0 | 0 | 0 | 0 | 0 | 0 | 0 | 0 |
| cv | 0 | 0 | 0 | 0 | 0 | 3 | 0 | 0 | 3 | 0 | 0 | 0 |
| t | 0 | 2 | 0 | 0 | 0 | 0 | 0 | 0 | 0 | 1 | 0 | 0 |
| i | 0 | 1 | 0 | 0 | 0 | 1 | 0 | 0 | 0 | 2 | 0 | 0 |
| ti | 0 | 1 | 0 | 0 | 0 | 1 | 0 | 0 | 0 | 2 | 0 | 0 |
| ah | 0 | 0 | 0 | 0 | 0 | 0 | 0 | 0 | 0 | 0 | 0 | 0 |
| mm | 0 | 0 | 0 | 0 | 0 | 0 | 0 | 0 | 0 | 0 | 0 | 0 |
| ptcml | 0 | 0 | 0 | 0 | 0 | 0 | 0 | 0 | 0 | 0 | 0 | 0 |

ABOi, ABO-incompatible; AMR, antibody-mediated rejection; HLAi, HLA-incompatible; mons, months; IF, immunofluorescence staining; IHC, immunohistochemical staining; NR, no rejection; TCMR, T cell-mediated rejection.

**TABLE S7.** Normalized gene expression counts across the samples

See a separate excel file.

**TABLE S8.** Functional enrichment analysis

See a separate excel file.

**

**

**FIGURE S1.** Regimen of an adjunctive eculizumab therapy and desensitization

ATG, thymoglobulin; D, day; Ec, eculizumab; IVIG, intravenous immunoglobulin; KT, kidney transplantation; W, week


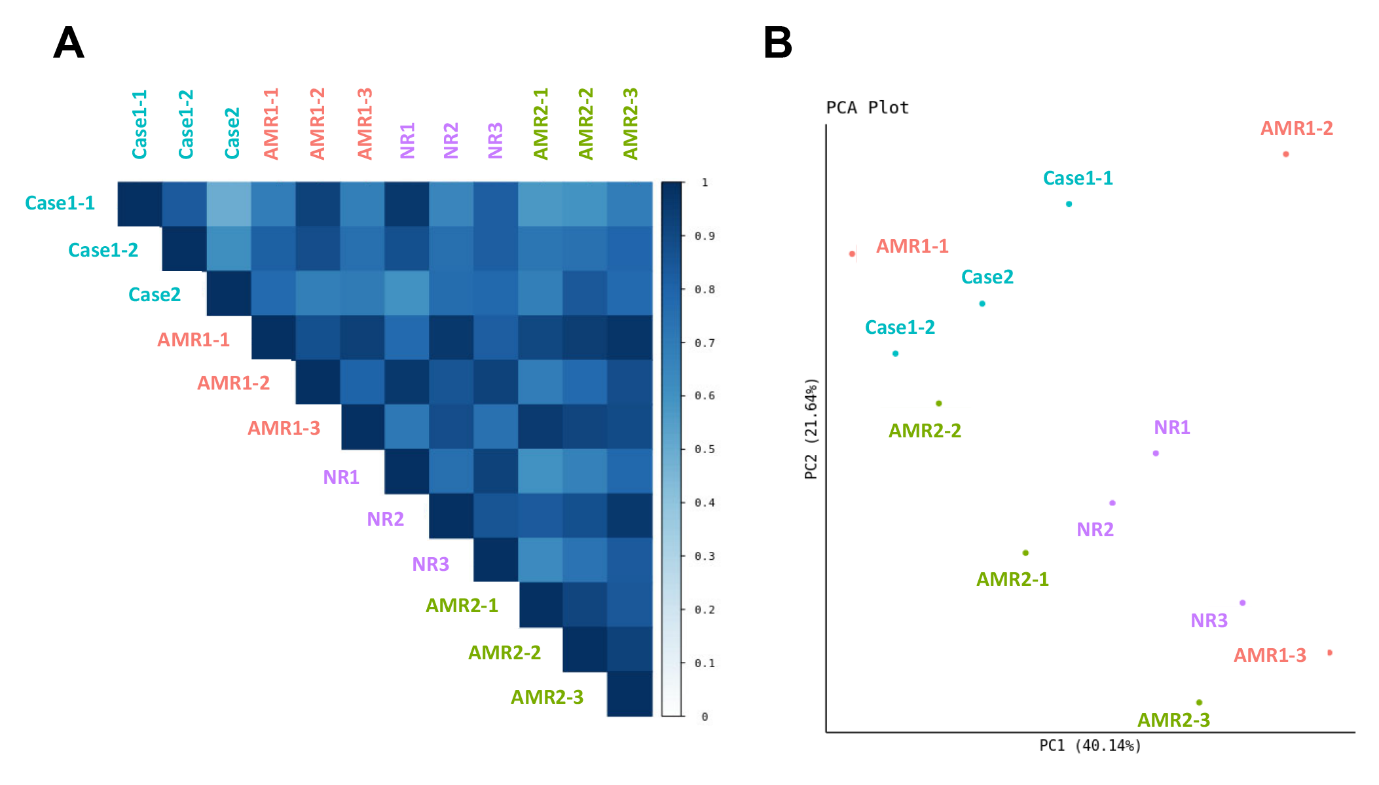


**FIGURE S2.** Correlation analysis and principal component analysis. (**a**) Pearson correlation coefficient between each sample subjected to B-HOT assay. (**b**) Principal component analysis plots of the samples. Note that biopsy samples 1-1 and 1-2 of case 1 do not exhibit notably stronger associations compared to the other biopsy samples.

AMR1, ABO-incompatible kidney transplantation group with antibody-mediated rejection; AMR2 ABO-incompatible and HLA-incompatible kidney transplantation group with antibody-mediated rejection; B-HOT, Banff Human Organ Transplant; Case, eculizumab group; NR, ABO-incompatible kidney transplantation group without antibody-mediated rejection; PCA, principal component analysis.


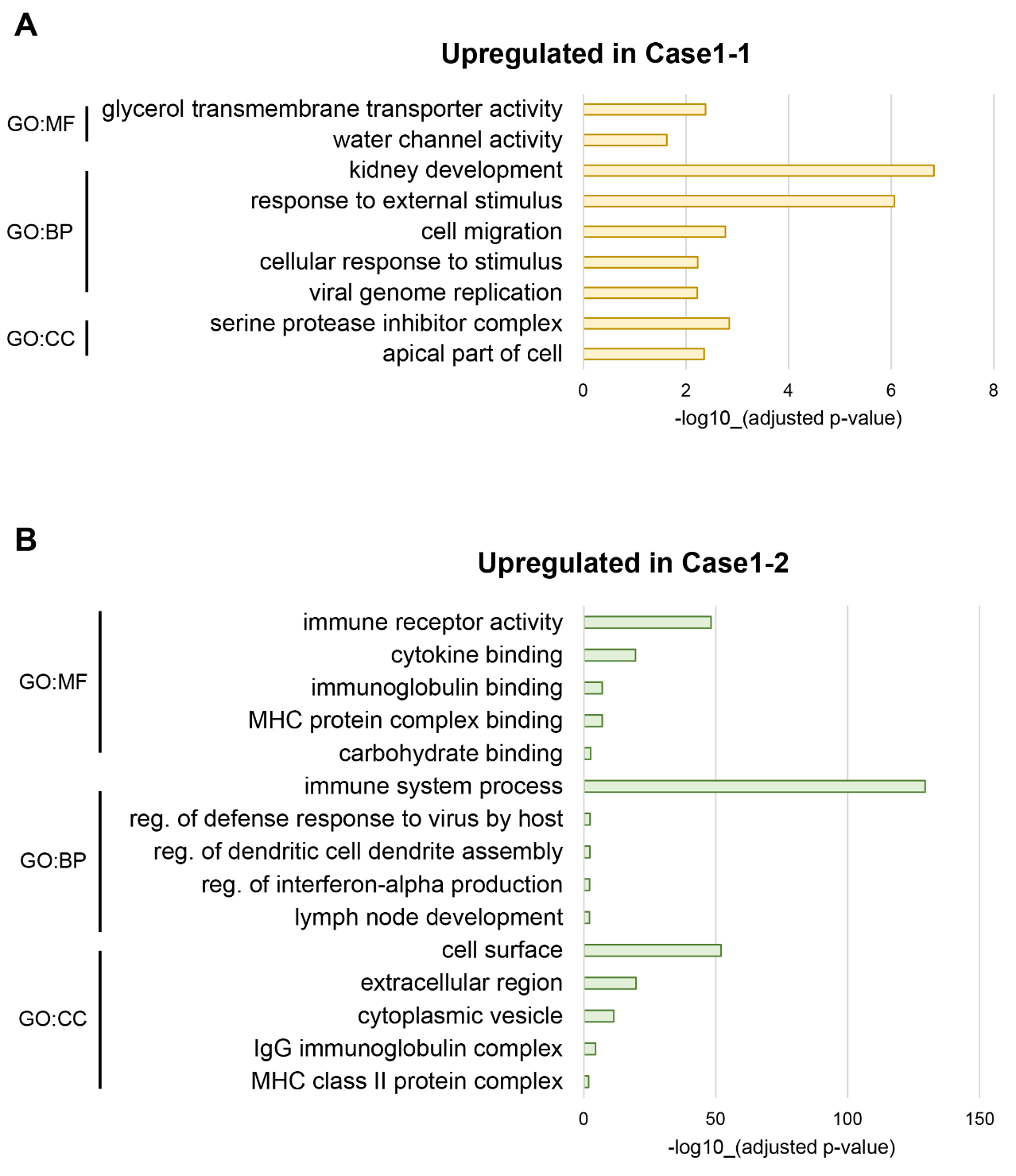


**FIGURE S3.** The top 5 Gene Ontology terms enriched in differentially expressed genes upregulated in the (**a**) first (biopsy 1-1) and (**b**) second (biopsy 1-2) biopsies of case 1. DEGs were determined at |fold-change| ≥ 2. GO-MF, GO-BP, and GO-CC terms were sorted based on the driver term function and adjusted *P* value in g:Profiler.

BP, biologic process; DEG, differentially expressed genes; GO, Gene Ontology; MF, molecular function; CC, cellular component.

**
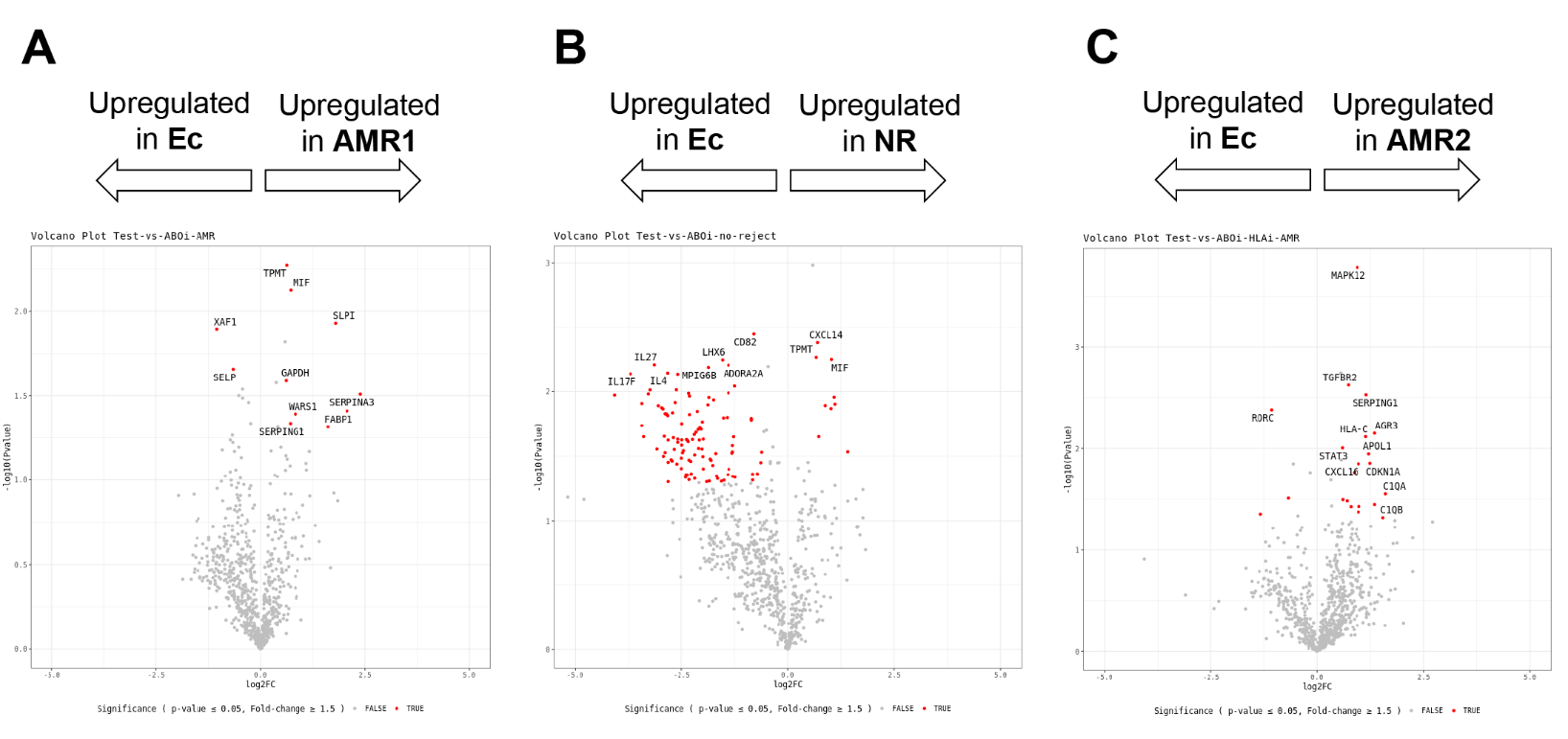
**

**FIGURE S4**. Differentially expressed genes between the (**a**) Ec and AMR1 groups, (**b**) Ec and NR groups, and (**c**) Ec and AMR2 groups

AMR1, ABO-incompatible kidney transplantation group with antibody-mediated rejection; AMR2 ABO-incompatible and HLA-incompatible kidney transplantation group with antibody-mediated rejection; Ec, eculizumab group; NR, ABO-incompatible kidney transplantation group without antibody-mediated rejection.

**
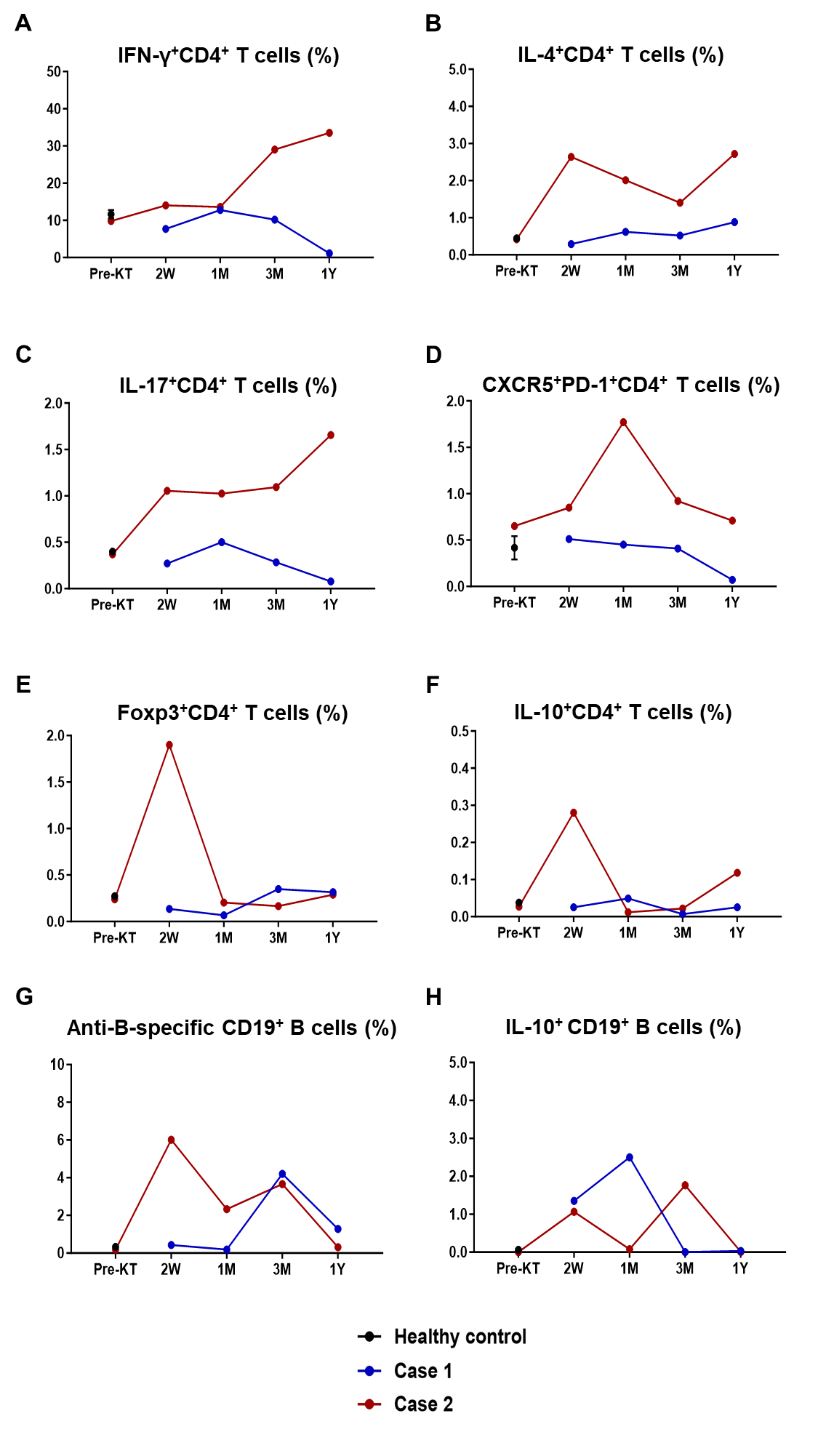
**

**FIGURE S5.** Changes in immune cell subsets during the post-transplant first year. Proportions of (**a**) IFN-γ^+^CD4^+^ T cells, (**b**) IL-4^+^CD4^+^ T cells, (**c**) IL-17^+^CD4^+^ T cells, (**d**) CXCR5^+^PD-1^+^CD4^+^ follicular helper T cells, (**e**) Foxp3^+^CD4^+^ regulatory T cells, (**f**) IL-10^+^CD4^+^ T cells, (**g**) anti-B-specific CD19^+^ B cells, and (**h**) IL-10^+^CD19^+^ B cells in the peripheral blood from the pre-KT period to one-year post-KT. Black, blue, and red circles indicate healthy controls, case 1, and case 2, respectively.

Foxp3, forkhead box P3; IFN, interferon; IL, interleukin; KT, kidney transplantation; M, month; PD-1, programmed cell death protein-1; W, weak; Y, year.
